# Determining the influence of the primary and specialist network of care on patient and system outcomes among patients with a new diagnosis of chronic obstructive pulmonary disease (COPD)

**DOI:** 10.1186/s12913-022-08588-w

**Published:** 2022-09-29

**Authors:** J. Tranmer, T. Rotter, D. O’Donnell, D. Marciniuk, M. Green, L. Kinsman, W. Li

**Affiliations:** 1 From the Department of Medicine, Family Medicine and Nursing ICES-Queen’s and Queen’s Health Services Policy Research Institute Queen’s Health Sciences, Queen’s University, Kingston, Canada; 2 Respiratory Research Center, University of Saskatchewan, Saskatoon, Canada; 3 School of Evidence Based Nursing, University of New Castle, New Castle, Australia

**Keywords:** Chronic obstructive pulmonary disease, Chronic disease management, Continuity of care

## Abstract

**Introduction:**

Care for patients with chronic obstructive pulmonary disease (COPD) is provided by both family physicians (FP) and specialists. Ideally, patients receive comprehensive and coordinated care from this provider team. The objectives for this study were**:** 1) to describe the family and specialist physician network of care for Ontario patients newly diagnosed with COPD and 2) to determine the associations between selected characteristics of the physician network and unplanned healthcare utilization.

**Methods:**

We conducted a retrospective cohort study using Ontario health administrative data housed at ICES (formerly the Institute for Clinical Evaluative Sciences). Ontario patients, ≥ 35 years, newly diagnosed with COPD were identified between 2005 and 2013. The FP and specialist network of care characteristics were described, and the relationship between selected characteristics (i.e., continuity of care) with unplanned healthcare utilization during the first 5 years after COPD diagnosis were determined in multivariate models.

**Results:**

Our cohort consisted of 450,837 patients, mean age 61.5 (SD 14.6) years. The FP was the predominant provider of care for 86.4% of the patients. Using the Bice-Boxerman’s Continuity of Care Index (COCI), a measure reflecting care across different providers, 227,082 (50.4%) were categorized in a low COCI group based on a median cut-off. In adjusted analyses, patients in the low COCI group were more likely to have a hospital admission (OR = 2.27, 95% CI 2.20,2.22), 30-day readmission (OR = 2.44, 95% CI 2.39, 2.49) and ER visit (OR = 2.27, 95% CI 2.25, 2.29).

**Conclusion:**

Higher indices of continuity of care are associated with reduced unplanned hospital use for patients with COPD. Primary care-based practice models to enhance continuity through coordination and integration of both primary and specialist care have the potential to enhance the health experience for patients with COPD and should be a health service planning priority.

## Introduction

Chronic obstructive pulmonary disease (COPD) is a chronic lung disease and contributes to the highest rates of hospital admissions among major chronic illnesses [1]. COPD is characterized by progressive, partially reversible airway obstruction and lung hyperinflation [2, 3] and is associated with shortness of breath, limitations in activities of daily living, decreased quality of life, and frequent exacerbations resulting in emergency visits and hospitalizations[3]. COPD is not well recognized by the general public and is frequently underdiagnosed [4–6]. Estimates for worldwide prevalence of COPD range from 4 to 20% [4]. Similar trends are reported in Canada, where the self-reported prevalence is around 4% [7] but more reliable estimates based on spirometry tests suggest a prevalence between 12 and 17% [4].

Despite the advances in medical treatment and guideline-based approaches, COPD exacerbations account for a substantial proportion of medical hospital admissions and emergent care [8], and are an ongoing concern. Given the complexity of pharmacological and non-pharmacological management, comprehensive medical management of COPD is key. Care is provided by both FP in the primary care setting and specialists, typically located in urban settings. Access to specialists is through a referral process. Ideally, the providers ensure patient access to the most appropriate level of care from this team of providers. However, barriers, such as geographical distance, specialist and FP access, and poor coordination, influence the provision of care.

It is assumed that continuity of care is enhanced when providers cohesively work together [9]. A patient-centric network is a constellation of providers (and services) that collaborate together to provide care to a common patient or groups of patients [10]. This network of providers and services will vary in number and ease of access. Recent evidence has clearly demonstrated geographical variation in distribution and access to primary and 24/7 care services for all patients in Ontario, the most populous province in Canada [11]. The impact of poor accessibility and availability is more relevant for different patient populations, such as adults with COPD. In Canada, health care is provided through territorial or provincial publicly funded systems. The provisions within the system are guided by the Canada Health Act of 1984 which supports universal access to publicly funded health (medical) services for all Canadians. In Ontario, within the past decade or so, the provincial ministry has introduced health care reforms to support the needs of the increasing proportion of persons with chronic health conditions.

Preventing fragmentation of care and enhancing continuity of care is a priority within these health care reforms. Continuity of care is multi-factorial and is often confused or used interchangeably with terms such as coordination of care, integration of care and seamless care [12]. Continuity of care is characterized by coherence of care across discrete events over time. Three types of continuity are often described: informational (e.g., flow of information), management (e.g., coherent services and care) and relational (e.g., patients and provider interactions). Continuity of care can be viewed from a provider or person perspective, however, for continuity of care to exist across different providers, conditions and sectors, it must be perceived as such by the patient. Indices or measures of continuity of care focus on duration of the patient-provider relationship, sequence of providers, dispersion of providers and subjective assessments [13]. In this paper we utilized three physician-claim based measures of continuity: Usual provider of care index (UPCI), continuity of care index (COCI), and sequential continuity of care index (SECONI). Detailed descriptions are provided in the methods section of this paper.

The overall aim of this study was to describe the network of specialist and family physician care for Ontario adult patients newly diagnosed with COPD. The specific research objectives were:

- 1. To describe the primary and specialist network of care of adult patients newly diagnosed with COPD in Ontario.
- 2. To determine the associations between selected network characteristics and unplanned healthcare utilization (i.e., hospital admissions, hospital readmission, emergency visits) in a 5-year window following COPD diagnosis, while controlling for potential confounders.

### Provisional hypothesis

We hypothesized that network characteristics (i.e., continuity of care) would be associated with less unplanned healthcare use, when controlling for confounders (age, sex, comorbidities, socioeconomic status, rurality).

## Methods

### Study design and setting

We conducted a retrospective cohort study, utilizing linked health administrative data housed at ICES (formerly known as the Institute for Clinical Evaluative Sciences. Ontario is the largest province in Canada (2020 population ~ 14,700,000) and has a coordinated single-payer healthcare delivery system. ICES is an independent, non-profit research organization primarily funded by the Ontario Ministry of Health and Long-Term Care (MOHLTC) whose comprehensive data holdings include all healthcare related events for the population of Ontario. All personal identifying information is removed at ICES, and an anonymous unique identifier, the ICES Key Number (IKN) is generated for each patient. The IKN is then used to link the databases at ICES. Ethics approval was obtained from the Health Sciences Research Ethics Board, Queen’s University, Kingston, Ontario, Canada.

### Data sources

The following linked administrative databases were used: (1) Canadian Institutes for Health Information, Discharge Abstract Data Base for information related to hospitalizations, (2) National Ambulatory Care Reporting System for information related to emergency visits, (3) Ontario Health Insurance Plan claims database for information related to physician billing, (4) Physician databases to identify specialists and family physicians, (5) Ontario Registered Persons Database for individual demographic information and (6) population census data for information related to geographical area dissemination codes.

### Study population

All patients aged 35 years or older with an initial new diagnosis date for COPD between April 1, 2005 and March 31, 2013 were identified in the ICES-derived COPD database. Patients in the ICES-derived cohort are identified using a case definition algorithm of 1 or more physician billing claims and/or 1 or more hospital discharges with a diagnosis of COPD as per the following codes: 91, 492, 496 (OHIP and *International Classification of Diseases, Ninth Revision* codes) or J41, J42, J43, J44 (*International Statistical Classification of Diseases, 10*^*th*^* Revision*codes). This COPD case definition algorithm was previously validated against a clinical reference standard in a population-based case verification study involving more than 400 individuals with and without COPD. In the verification study, the case definition algorithm had a sensitivity of 85.0% and a specificity of 78.4% [14]. Once patients were identified as having COPD, they were followed for 5 years, unless they died or moved out of Ontario. Exclusion criteria were: (1) an invalid IKN (patient identifier), (2) non-Ontario residents, (3) no Ontario Health Insurance (OHIP) coverage for the entirety of the study, and (4) a history of COPD diagnosis before April 1, 2005.

### Network characteristics

For patients in the cohort, we identified the physician specialty using the ICES Physician database (IPDB) of OHIP claims during the study period. Physician’s specialty was categorized as: (1) Family Physician (FP), and (2) Respiratory specialist (pediatric respirology, respirology and internal medicine). We did not include the OHIP billing claims of other specialists (i.e., cardiology). Patients were then categorized patients into predominant provider groups, based on the proportion of visits to either the FP or specialist group. Please note that this is different than the Usual Provider Care Index (UPCI), as outlined below.

We defined 3 continuity measures within the physician network of care [15].

1. *Usual provider of care index (UPCI)* is a measure of longitudinal continuity defined as the proportion of patient visits to their main provider to the total number patient visits for all providers of interest. Values range from 0 (no main provider) to 1 (same provider).
2. *Continuity of care* as measured with the Bice-Boxerman Concentration of Care Index (COCI) is an expression of the dispersion of all physician visits over time across the number of individual physicians visited [16]. The COCI is sensitive to changes in the total number of visits and their distribution across different providers, with a score of 0 occurring when each visit is to a different provider and 1 when all visits are to the same provider.
3. *Sequential continuity of care index (SECONI)* measures the sequential nature of provider continuity, is the fraction of sequential visits and is dependent upon the order of visits. There is a similar range of 0 to 1, with 0 reflecting an alternation between 2 providers and 1 reflecting sequential visits to the same provider.

We also determined the *geographical distance* as measured distance from patient’s residence to the usual provider of care. Distance was calculated with the “best estimated” geolocations (latitude-longitude) between the patient’s postal code at index date and physician’s main billing office in the IPDB database.

### Unplanned healthcare utilization

Our definition of unplanned healthcare utilization included ED visits without hospital admission, ED visits with a hospital admission (not scheduled and excluded transfers), any hospital admission and 30-day readmissions.

### Confounding variables

All models controlled for age, sex, comorbidity as measured with the John Hopkins Diagnostic Grouping [17, 18] and specific comorbid conditions, and socioeconomic status as measured by geographical dissemination area descriptors and the Ontario Marginalization Index [19] and rurality.

### Analyses

We initially described, using standard univariate statistics, the patient population and the patients’ care experience with a specific focus on network characteristics [20]. Comparison of patient characteristics between FP and specialist groups and low and high COCI categories were determined, and reported as standardized differences. Standardized difference of < 0.1 reflects no difference in means or distribution between the FP and specialist groups. Logistic regression models were used to estimate the odds ratio (OR) between COCI category (defined by the median cut-off score) and each study outcome (ER visits, hospital admission and readmission). Unadjusted associations were estimated, and models were then adjusted for confounding factors of age, sex, rurality, comorbidity conditions, and social status (Model A) and physician group (Model B).

## Results

The inception cohort included 450,837 adult (≥ 35 years) Ontario community-dwelling residents, with a new diagnosis of COPD during the 5-year recruitment window (2008–2013). We included a 2-year look-back window to identify comorbidities. Patients were followed until March 31, 2018. At cohort conception the mean age was 61.5 (SD 14.6) years, 50% were female, with 64.6% residing in lower 3 income quintile areas, and 84.6% residing in urban locations. Sixty-eight per cent had more than 4 comorbidities. We compared the population characteristics with the overall Ontario population, ≥ 35 years, in 2010; with no unexpected differences. See Table 1.

**Table 1:** Characteristics of the patient cohort and comparable Ontario population

		**FP (*n* = 425,070)**	**Specialist (*n* = 25,767)**	**Ontario population (*n* = 7,272,600)**
**Demographics**				
Age (years)	Mean ± SD	61.24 ± 14.61	66.03 ± 14.41	55.78 ± 13.67
	Median (IQR)	60 (50–73)	66 (55–78)	53 (45–65)
Age Group	31–40	33,357 (7.8%)	1,098 (4.3%)	941,598 (12.9%)
	41–50	82,391 (19.4%)	3,154 (12.2%)	2,116,360 (29.1%)
	51–64	135,024 (31.8%)	7,472 (29.0%)	2,380,291 (32.7%)
	65 +	174,289 (41.0%)	14,043 (54.5%)	1,834,351 (25.2%)
Sex	F	213,544 (50.2%)	10,757 (41.7%)	3,778,781 (52.0%)
	M	211,517 (49.8%)	15,010 (58.3%)	3,493,819 (48.0%)
Dependency	Mean ± SD	3.25 ± 1.43	3.27 ± 1.43	2.99 ± 1.45
Material deprivation	Mean ± SD	3.22 ± 1.41	3.31 ± 1.43	3.06 ± 1.42
Ethnic concentration	Mean ± SD	2.87 ± 1.43	3.04 ± 1.43	3.17 ± 1.44
Residential instability	Mean ± SD	3.32 ± 1.38	3.48 ± 1.39	3.03 ± 1.44
Ontario marginalization index	Mean ± SD	3.16 ± 0.78	3.28 ± 0.79	3.06 ± 0.77
Income quintile	1 (lowest)	97,662 (23.1%)	6,633 (25.9%)	1,329,933 (18.3%)
	2	91,750 (21.7%)	5,572 (21.7%)	1,417,938 (19.5%)
	3	83,793 (19.8%)	4,748 (18.5%)	1,442,931 (19.8%)
	4	79,550 (18.8%)	4,512 (17.6%)	1,526,134 (21.0%)
	5 (highest)	70,649 (16.7%)	4,155 (16.2%)	1,530,451 (21.0%)
Rural status	N	358,403 (84.3%)	22,872 (88.8%)	6,370,810 (87.6%)
	Y	66,600 (15.7%)	2,889 (11.2%)	898,174 (12.4%)
**Clinical characteristics**				
ADG comorbidity Score	1–4	136,715 (32.2%)	6,442 (25.0%)	3,836,306 (52.8%)
	5–9	213,332 (50.2%)	11,374 (44.1%)	2,043,275 (28.1%)
	10 +	75,023 (17.6%)	7,951 (30.9%)	315,384 (4.3%)
Asthma	Yes	86,898 (20.4%)	5,002 (19.4%)	800,276 (11.0%)
Diabetes	Yes	78,340 (18.4%)	6,605 (25.6%)	1,058,359 (14.6%)
Hypertension	Yes	205,504 (48.3%)	14,145 (54.9%)	2,583,329 (35.5%)

• Dependency refers to area-level concentrations of persons who do not have income from employment; indicator measures include seniors, children and adults with non-compensated work

• Material deprivation is related to the inability of individuals or communities to access and attain basic material needs; indicator measures include income, quality of housing, educational attainment and family structure characteristics

• Ethnic concentrations refers to area-level concentration of recent immigrants and people, other than aboriginal peoples, who are non-Caucasian in race or non-white in colour

•Residential instability refers to area-level concentrations of people who experience high rates of family or housing instability; indicator measures include types and density of residential accommodations and family structure characteristics

• Ontario marginalization index is a composite index of the dimensions, with a score ranging from 1–5, with 1 representing low levels of marginalization and 5 representing high levels of marginalization

• The Ontario population characteristics are derived from PCOP, a population level dataset that includes all people in Ontario who are deemed alive and eligible at a given point in time. All indicators are as of the date March 31, 2010, among persons > 35 years of age, with various look back periods

### Network of care

The FP was identified as the predominant provider of care (i.e., the highest proportion of visits were to the FP) for most patients (*n* = 425,070, 94.2%) and most patients had 20 or more visits over the 5 years. The UPCI score which reflects the density of care was higher among patients categorized with the FP as the main provider: UPCI score for FP was 0.60 (SD 0.22) and for specialists 0.39 (SD 0.18). Whereas the COCI scores, reflecting the continuity of care, were similar for both the FP and specialist provider groups; the overall mean COCI score was 0.51 (SD 0.26). The SECONI scores, reflecting sequential continuity of visits were slightly higher within specialist versus usual provider group. Patients travelled further to see their specialist provider. See Table 2.

**Table 2:** Physician network characteristics

Number of patients		**All**	**Family Physician**	**Specialist**	**Standardized difference**
		*N* = 450,837	*N* = 425,070	*N* = 25,767	
Outpatient Visits for 5 years	1–5	4,729 (1.0%)	2,433 (0.6%)	2,296 (8.9%)	0.64
	6–10	37,773 (8.4%)	32,899 (7.7%)	4,874 (18.9%)	
	11–20	88,456 (19.6%)	81,544 (19.2%)	6,912 (26.8%)	
	20 +	319,879 (71.0%)	308,194 (72.5%)	11,685 (45.3%)	
Mean Proportion of FP or SP visits/total visits			86.4%	13.6%	
Usual provider of care index (UPCI)	Mean ± SD	0.59 ± 0.22	0.60 ± 0.22	0.39 ± 0.18	1.01
	Median (IQR)	0.59 (0.41–0.77)	0.60 (0.43–0.78)	0.36 (0.26–0.50)	1.02
Continuity of care index (COCI)	Mean ± SD	0.51 ± 0.26	0.52 ± 0.26	0.51 ± 0.29	0.02
	Median (IQR)	0.49 (0.30–0.73)	0.49 (0.30–0.73)	0.45 (0.27–0.74)	0.04
Sequential continuity of care index (SECONI)	Mean ± SD	0.64 ± 0.23	0.64 ± 0.22	0.68 ± 0.25	0.16
	Median (IQR)	0.67 (0.50–0.82)	0.67 (0.45–0.81)	0.71 (0.50–0.87)	0.18
High or low COCI (based on median cutoff)	High COCI	223,755 (49.6%)	212,202 (49.9%)	11,553 (44.8%)	0.1
	Low COCI	227,082 (50.4%)	212,868 (50.1%)	14,214 (55.2%)	
Distance to Usual provider(km)	Mean ± SD	17.55 ± 56.51	17.08 ± 55.12	25.32 ± 75.45	0.12
	Median (IQR)	5 (2–14)	5 (2–14)	6 (3–18)	0.21

*Note*: FP and Specialist categories are based on the highest proportion of visits to each provider. Continuity of Care Measures were determined using billing codes for any outpatient physician visits (including FP and specialty care visits) over the 5-year observation window and excluding participants who had few visits (< = 5).**Usual Provider Care (UPCI):** UPCI reflects the concentration of care with a single provider or group of providers across time. UPC is measured by using the highest number of visits to a single practitioner or a group of practitioner and then divided by the total number of visits**. Bice-Boxerman’s Concentration of Care Index (COCI):** This measure captures the care across different providers as well as the care coordination back to the family physician. **Sequential Continuity of Care (SECONI)**: SECONI considers the order of the visits and captures the number of handoffs between providers

### Healthcare utilization

Overall, at least half of the sample had a hospital admission over 5 years, for any reason. Healthcare utilization varied between the designated provider groups. Patients cared for by FPs had fewer hospital admissions (all cause and COPD related), readmissions and ER visits. See Table 3. Healthcare utilization also varied between patients identified within the low vs high COCI categories. Patients in the low COCI categories had higher unplanned healthcare utilization and were more likely to die during hospitalization. See Table 4.

**Table 3:** Health care utilization during the 5 years of follow up: family physician and specialist as the usual provider of care

Number of COPD patients		**FP**	**Spec**	**Standardized Difference**
		*N* = 425,070	*N* = 25,767	
Number of hospital admissions (any cause)	0	237,207 (55.8%)	9,107 (35.3%)	0.47
	1 to 5	174,968 (41.2%)	14,979 (58.1%)	
	6 to 10	10,959 (2.6%)	1,294 (5.0%)	
	> 10	1,936 (0.5%)	387 (1.5%)	
Mean number of hospital admissions per patient		1.03 ± 1.83	1.77 ± 2.61	0.33
Number hospital admissions (COPD related)	0	378,120 (89.0%)	19,744 (76.6%)	0.34
	1 to 5	45,677 (10.7%)	5,724 (22.2%)	
	6 to 10	1,116 (0.3%)	247 (1.0%)	
	> 10	157 (0.0%)	52 (0.2%)	
Mean number of hospital admission per patient (COPD related)		0.19 ± 0.74	0.47 ± 1.31	0.26
Discharge due to death		30,457 (7.2%)	6,790 (26.4%)	0.53
30 day readmission		35,600 (8.4%)	4,229 (16.4%)	0.25
Number of ER visits (any cause)	0	99,176 (23.3%)	6,044 (23.5%)	0.04
	1 to 5	234,998 (55.3%)	14,044 (54.5%)	
	6 to 10	57,157 (13.4%)	3,480 (13.5%)	
	> 10	33,739 (7.9%)	2,199 (8.5%)	
Number of ER visits (COPD related)	0	383,582 (90.2%)	21,625 (83.9%)	0.27
	1 to 5	39,688 (9.3%)	3,839 (14.9%)	
	6 to 10	1,404 (0.3%)	208 (0.8%)	
	> 10	396 (0.1%)	95 (0.4%)	
Number of ER visits with hospital admission	0	278,936 (65.6%)	11,272 (43.7%)	0.47
	1 to 5	136,917 (32.2%)	13,216 (51.3%)	
	6 to 10	7,748 (1.8%)	992 (3.8%)	
	> 10	1,469 (0.3%)	287 (1.1%)

**Table 4:** Health care utilization during the 5 years of follow up: between patients with low vs high COC scores

Number of COPD patients		All		Standardized difference
		**Low COCI**	High COCI*	
		*N* = 227,082	*N* = 223,755	
Number of hospital admissions (any cause)	0	106,548 (46.9%)	139,766 (62.5%)	0.34
	1 to 5	108,796 (47.9%)	81,151 (36.3%)	
	6 to 10	9,702 (4.3%)	2,551 (1.1%)	
	> 10	2,036 (0.9%)	287 (0.1%)	
Mean number of hospital admissions per patient		1.42 ± 2.27	0.72 ± 1.32	0.38
Number hospital admissions (COPD related)	0	192,655 (84.8%)	205,209 (91.7%)	0.22
	1 to 5	33,071 (14.6%)	18,330 (8.2%)	
	6 to 10	1,170 (0.5%)	193 (0.1%)	
	> 10	186 (0.1%)	23 (0.0%)	
Mean number of hospital admission per patient (COPD related)		0.29 ± 0.97	0.12 ± 0.52	0.21
Discharge due to death		24,402 (10.7%)	12,845 (5.7%)	0.18
30 day readmission		28,547 (12.6%)	11,282 (5.0%)	0.27
Number of ER visits (any cause)	0	35,222 (15.5%)	69,998 (31.3%)	0.53
	1 to 5	121,728 (53.6%)	127,314 (56.9%)	
	6 to 10	41,059 (18.1%)	19,578 (8.7%)	
	> 10	29,073 (12.8%)	6,865 (3.1%)	
Number of ER visits (COPD related)	0	197,352 (86.9%)	207,855 (92.9%)	0.23
	1 to 5	27,924 (12.3%)	15,603 (7.0%)	
	6 to 10	1,368 (0.6%)	244 (0.1%)	
	> 10	438 (0.2%)	53 (0.0%)	
Number of ER visits with hospital admission	0	126,031 (55.5%)	164,177 (73.4%)	0.37
	1 to 5	92,249 (40.6%)	57,884 (25.9%)	
	6 to 10	7,213 (3.2%)	1,527 (0.7%)	
	> 10	1,589 (0.7%)	167 (0.1%)	

• All comparisons were significantly different

### Influence of continuity of care

As reported in Table 5, in adjusted analyses that included relevant sociodemographic characteristics, patients in the low COCI care category were more likely to have hospital admissions (OR = 2.27, 95% CI = 2.24,2.29), emergency visits (OR = 2.28, 95% CI = 2.27,2.30), any 30 day readmission (OR = 2.45, 95% CI = 2.40,2.50) and greater number of readmissions (OR = 3.68, 95% CI = 3.58,3.78). These OR effect estimates did not change with the addition of physician provider group in the model.

**Table 5:** Association between continuity of care index and health care utilization

	**Unadjusted OR**	**95% CI**	**Adjusted OR**^**a**^	**95%C I**	**Adjusted OR**^**b**^	**95%C I**
Hospital admission	2.40	2.37,2.43	2.27	2.24,2.29	2.20	2.17,2.22
Emergency visits	2.47	2.45,2.49	2.28	2.27,2.30	2.27	2.25,2.29
Any 30 day readmission	2.49	2.44,2.55	2.45	2.40,2.50	2.44	2.39,2.49
Number of readmissions	3.95	3.84,4.06	3.68	3.58,3.78	3.51	3.42,3.60

*Note*: High COC is the reference category

Model A: Adjusted for age, sex, comorbidities(ADG), SES, rurality

Model B: Adjusted for Model A covariates plus provider group

## Discussion

We conducted a retrospective cohort study of Ontario persons who were classified with a new diagnosis of COPD, between 2005 and 2013. Patients were followed for 5 years following their new diagnosis of COPD. As expected, patients were predominantly older adults who had other comorbidities along with their new COPD diagnoses. Healthcare utilization was high in the first 5 years following diagnoses, with most having at least one hospital admission and/or emergency visit. Unfortunately, this pattern of healthcare utilization for patients with COPD is common.

As reported previously, the primary care provider is the predominant provider of care for patients with COPD [21]. Patients visited their primary care provider often; with most having more than 20 visits over the 5 years. The COCI for primary care and specialist providers was similar with a mean COCI score 0.5, somewhat lower than COCI reported in similar studies exploring the effects of continuity of care on healthcare use among COPD patients in Korea [22] and Taiwan [23]. COCI scores can be influenced by the number of providers within the network of care or the ability of the patient to access different providers, through services such as walk-in-clinics or emergency. While the sociodemographic and descriptive patient characteristics were relatively similar between the specialist and provider groups; patients who received care predominantly from the specialist had higher rates of unplanned healthcare utilization, and death during hospitalization. These higher rates are logical if we assume that this group of patients had more severe disease (which we were not able to measure) that required hospitalization and required specialist care. This is reassuring as it suggests that specialist care and services are being provided to patients with more advanced disease.

In this study, lower COCI scores, regardless of family physician or specialty designation, were consistently associated with worse outcomes. Patients classified within the lower COCI category were twice as likely to have hospital admissions, emergency visits and readmissions. These patients were more likely to die during a hospitalization. These worse outcomes persisted when other personal, social and clinical factors were considered. Continuity of care reflects the degree to which care events experienced by people is coherent and interconnected over time and reflects relational continuity with the primary care provider or team. Continuity of care occurs when there is coordinated and seamless interactions among multiple providers. In this study we used 3 claims based measured of continuity of care. The UPCI reflects the density of care, and as expected FPs provided a greater proportion of care. The UPCI measure does not account for the number of providers seen nor the distribution of visits to other providers which is common for patients with COPD who often need care from a variety of providers. The COCI is sensitive to increasing number of providers as the measure reflects management continuity and assumes that repeated visits reflect a stable relationship. In our analysis the COCI was similar in both FP and specialist groups. If we assume patients with more severe illness may need specialist care, this finding is reassuring as it reflects that when the specialist is the predominant provider of care they are able to provide cohesive specialist care. The SECONI reflects the sequencing of visits. Our finding that the SECONI was slightly higher within the specialist group could be related to the nature of COPD management, that is, intense, cohesive care during exacerbations. While these are all measures of longitudinal continuity, they do not capture the multi-dimensionality of continuity of care, as previously described. Future research should also address the other aspects of continuity such as information exchange and interpersonal relations. However, our findings are consistent with other studies that support the need to foster continuity of care strategies to improve outcomes [24, 25].

Within the context of healthcare reforms, continuity of care improvement strategies can take many forms. For example, recent Ontario reforms are directed towards the creation of Ontario Health Teams (OHT). OHT are groups of healthcare providers and organization that are clinically and fiscally accountable for delivering a full coordinated continuum of care to a defined geographic population [26], similar to accountable care organizations developed within the USA [27]. We would strongly suggest that attachment or rostering of a patient to a primary care provider is a key component of effective coordinated care. Other key components, common to the management of other chronic conditions, include: team based approach [28], timely access to specialty consultations and diagnostic services, guideline based care or pathways [27, 29, 30], access to pulmonary rehabilitation [31], self-management supports [32, 33]; all recommendations outlined in current guidelines [2, 34].

There are barriers and facilitators for provision of coordinated primary care based, specialty supported care for COPD patients. Concerns are often raised about access to and availability of specialist services. In Ontario, the reported proportion of respirologists to the population is 2.1/100,000 population (CMA Workforce Survey, Respirology Profile, 2019), similar to the Canadian proportion of 2.2/100,000 population. Approximately half of the respirologists report working at Academic Health Sciences Centres, within urban centres. Moreover, only a proportion of these respirologists focus on providing care and follow-up for patients with COPD. Thus, strategies to further optimize respirology care and management are important. Organizational strategies such as multi-professional pulmonary COPD clinics are designed to provide comprehensive care and management, optimize healthcare provider roles and are associated with positive clinical outcomes [35]. Unfortunately, availability and access to these services is also limited. Primary care or community-based COPD rehabilitation clinics offer promise and address issues related to access and availability of services [36–38]. Patient-centric reforms, such as those targeted within the OHT reforms, should provide a platform for integration of “wrap around” services for the patient and optimize the utilization of existing resources. Findings from our study support the benefits of a continuity of care approach.

There are limitations to this retrospective cohort study. First, COPD classification of persons was based on physician billing and coding, and not detailed diagnostic criteria (i.e., spirometry testing). While the algorithm has a reported sensitivity of 85%, when compared to chart abstraction, we are likely underestimating the prevalence of COPD in the general population. Moreover, it seems that we are capturing patients at a later stage in the diagnostic window, as reflected in the number of hospitalizations and deaths. As previously stated, we did not capture disease severity or progression during the follow-up period. While disease severity certainly influences hospitalizations and death, our analysis which focused on the provision of both specialist and FP care found that regardless of disease state patients did better when there was higher continuity of care. Second, the physician network characteristics were limited to the inclusion of primary care and respirology physicians. We know that patients with chronic conditions such as COPD are seen by other physician specialists [21], which may further influence the continuity of care. Furthermore, we did not account for physicians who work collaboratively within a group or team model. Each visit within the group would be allocated to the individual physician and not the group. Our cohort only included Ontario patients; however, our findings are likely generalizable to other provincial jurisdictions and countries with similar healthcare systems. Finally, we did not capture the organizational processes and structures that would influence the provision of care for patients with COPD. Nevertheless, findings from this study do show that newly diagnosed patients do benefit from continuous and cohesive care.

## Conclusion

Continuity of care for patients with COPD is associated with reduced unplanned healthcare use for patients with newly diagnosed with COPD. Continuity of care characterized by practice models that enhance coordination and integration of primary and specialists services care have the potential to positively impact the health experience for patients with COPD and should be a health service planning priority.

## Data Availability

The dataset from this study is held securely in coded form at ICES. While data sharing agreements prohibit ICES from making the dataset publicly available, access may be granted to those who meet pre-specified criteria for confidential access, available at www.ices.on.ca/DAS. The full dataset creation plan and underlying analytic code are available from the author JT upon request, understanding that the computer programs may rely upon coding templates or macros that are unique to ICES and are therefore either inaccessible or may require modification.

## Abbreviations

COPD: Chronic obstructive pulmonary disease
COCI: Bice Boxerman’s continuity of care index
SECONI: Sequential continuity of care index
UPCI: Usual provider care index
OHIP: Ontario health insurance plan
FP: Family physician
OHT: Ontario health team
